# Plasma miRNA Profiles in Pregnant Women Predict Infant Outcomes following Prenatal Alcohol Exposure

**DOI:** 10.1371/journal.pone.0165081

**Published:** 2016-11-09

**Authors:** Sridevi Balaraman, Jordan J. Schafer, Alexander M. Tseng, Wladimir Wertelecki, Lyubov Yevtushok, Natalya Zymak-Zakutnya, Christina D. Chambers, Rajesh C. Miranda

**Affiliations:** 1 Department of Neuroscience and Experimental Therapeutics, Texas A&M Health Science Center, College of Medicine, Bryan, Texas, United States of America; 2 Department of Pediatrics, University of California San Diego, La Jolla, California, United States of America; 3 Omni-Net Ukraine Birth Defects Prevention Program, Rivne, Ukraine; 4 Rivne Provincial Medical Diagnostic Center and OMNI-Net Center, Rivne, Ukraine; 5 Khmelnytsky City Perinatal Center and OMNI-Net Center, Khmelnytsky, Ukraine; 6 Department of Family Medicine and Public Health, University of California San Diego, La Jolla, California, United States of America; 7 The Collaborative Initiative on Fetal Alcohol Spectrum Disorders (CIFASD), San Diego, California, United States of America; Oregon Health and Science University, UNITED STATES

## Abstract

Fetal alcohol spectrum disorders (FASD) are difficult to diagnose since many heavily exposed infants, at risk for intellectual disability, do not exhibit craniofacial dysmorphology or growth deficits. Consequently, there is a need for biomarkers that predict disability. In both animal models and human studies, alcohol exposure during pregnancy resulted in significant alterations in circulating microRNAs (miRNAs) in maternal blood. In the current study, we asked if changes in plasma miRNAs in alcohol-exposed pregnant mothers, either alone or in conjunction with other clinical variables, could predict infant outcomes. Sixty-eight pregnant women at two perinatal care clinics in western Ukraine were recruited into the study. Detailed health and alcohol consumption histories, and 2^nd^ and 3^rd^ trimester blood samples were obtained. Birth cohort infants were assessed by a geneticist and classified as unexposed (UE), heavily prenatally exposed and affected (HEa) or heavily exposed but apparently unaffected (HEua). MiRNAs were assessed in plasma samples using qRT-PCR arrays. ANOVA models identified 11 miRNAs that were all significantly elevated in maternal plasma from the HEa group relative to HEua and UE groups. In a random forest analysis classification model, a combination of high variance miRNAs, smoking history and socioeconomic status classified membership in HEa and UE groups, with a misclassification rate of 13%. The RFA model also classified 17% of the HEua group as UE-like, whereas 83% were HEa-like, at least at one stage of pregnancy. Collectively our data indicate that maternal plasma miRNAs predict infant outcomes, and may be useful to classify difficult-to-diagnose FASD subpopulations.

## Introduction

Fetal Alcohol Spectrum Disorders (FASD) are a leading cause of intellectual disability in the US and worldwide. The global prevalence of Fetal Alcohol Syndrome (FAS), the severe end of the FASD continuum, is estimated at ~2.9‰, with regional prevalence estimates ranging up to 55.42‰, and the prevalence for FASD at 22.77‰, with regional highs of up to 113.22‰ [1]. FASD-associated cognitive and behavioral deficits (reviewed in [2]) contribute to the emergence of secondary mental health disabilities [3, 4], and result in significant public health and economic burdens [5].

Despite published prevention guidelines [6], FASD remains difficult to prevent, because women with unplanned pregnancies, ~53% of pregnancies in US women aged 30 years and younger [7], may not recognize their pregnancy status and episodically engage in heavy drinking including binge drinking [8], a pattern that is particularly damaging to fetal development [9]. There is therefore, a significant need to identify affected children early, to facilitate early intervention, and mitigate disabilities that emerge later in life [10]. However, early diagnosis is difficult, because while some heavily exposed infants exhibit distinctive facial dysmorphology, small head-circumference and perinatal growth restriction [11], other heavily exposed children with neurodevelopmental impairment do not exhibit readily identifiable dysmorphic features [12, 13] associated with FASD. Moreover, a documented history of drinking during pregnancy is often difficult to ascertain [14]. Previous studies have identified ethanol metabolites in neonatal meconium [15, 16], placenta [17], and in newborn dried blood spots [18] as biomarkers for fetal alcohol exposure. However, these biomarkers are not specifically predictors of infant health outcomes. Here we assessed microRNAs (miRNAs) that are present in maternal plasma during pregnancy as potential predictors of infant outcomes, following prenatal alcohol exposure.

MiRNAs are small non-protein-coding RNA molecules that repress protein translation. In 2007, we showed that miRNAs were sensitive to alcohol [19] and mediated alcohol effects on fetal neural [19, 20] and cranial development [21] in animal models. MiRNAs play an important role in alcohol addiction [22–26] and neurotoxicity [27], alcohol-associated alterations in intestinal and hepatic integrity [28, 29], inflammation [30], fibrosis [31], and bone remodeling [32]. Thus, miRNAs are not only sensitive to alcohol exposure, but also mediate many alcohol effects.

In 2008, evidence emerged that miRNAs were secreted into plasma [33] and could be used to diagnose disease [34, 35]. Many organs are predicted to secrete miRNAs into circulation [36], however, during pregnancy fetal tissues like the placenta are an additional source of miRNAs in maternal circulation [37]. Consequently, maternal plasma miRNAs have a dual origin and may therefore serve as composite biomarkers for both maternal and fetal health. We found that ethanol exposure in an ovine pregnancy model, altered maternal plasma miRNAs [38]. Recently, exposure during pregnancy in humans has also been shown to alter maternal serum miRNA content [39]. These data collectively show that circulating miRNAs, like ethanol metabolites, may be used as biomarkers for exposure to alcohol exposure during pregnancy. However, the question that arises is, “*can circulating miRNAs in the pregnant mother predict infant outcomes*?” To address this question, we assessed the relationship between miRNAs in plasma samples obtained from pregnant women who reported varying levels of alcohol consumption, to the presence or absence of FASD characteristics in their offspring.

## Materials & Methods

### Description of the Cohort

The sample for this study was drawn from a larger group of pregnant women and their infants who were enrolled in a longitudinal cohort study conducted in two regions of Western Ukraine as part of the Collaborative Initiative on Fetal Alcohol Spectrum Disorders (CIFASD.org) between the years 2006 and 2011. The sites, a prenatal diagnostic center in the Rivne province and a perinatal center in the Khmelnytsky province where women received prenatal care, were members of the Omni-Net Ukraine Birth Defects Prevention Program. Recruitment methods have been described in detail elsewhere [40, 41]. Briefly, pregnant women were screened for alcohol consumption and selected for enrollment if they reported either frequent daily or weekly episodic (binge) drinking in the month around conception or the most recent month of pregnancy. For each enrolled woman who met the frequent or heavy drinking criteria, the next eligible woman who reported infrequent or no alcohol consumption and no binge drinking was also asked to participate. At enrollment, women provided written informed consent, and were interviewed extensively about demographics, pregnancy and health history, tobacco and other drug use. Women were asked about day-by-day alcohol consumption in the week around conception (variables AAD0 and AADD0, Table 1) and the most recent two weeks of pregnancy (variables AADXP and AADDXP, Table 1) using the timeline followback procedure [42]. The timeline follow back procedure generates day-by-day quantity and frequency estimates of past alcohol and drug consumption with high test-retest reliability [43], and has been validated for assessment of past alcohol use in populations of pregnant women [44]. Women were enrolled in the study on average between 17 and 19 weeks of pregnancy (Table 1), and therefore, the AADXP and AADDXP variables recorded alcohol consumption in the previous two weeks, during the second trimester. Women were interviewed again in the third trimester about alcohol consumption. Patient gestational age at enrollement, alcohol consumption estimates and other demographic and clinical characteristics are outlined in Table 1. Blood samples were collected by venipuncture, into EDTA-coated tubes, from mothers at each of the two interview timepoints, and centrifuged. Aspirated plasma samples were frozen, shipped to investigators in the U.S. and stored at -80°C until analysis.

**Table 1:** Maternal characteristics of the sample, Ukraine, 2006–2011.

Variable		HEa (N = 22)	HEua (N = 23)	UE (N = 23)	p-value
Mother’s age at enrollment (*momage*)		26.95 ±6.00	24.04 ± 4.40	26.30 ± 4.70	0.1377^a^
Gestational age at enrollment		18.60 ± 4.83	19.13 ± 5.90	17.94 ± 6.38	0.5051^a^
Recruitment site	Khmelnytsky	50.0% (11)	17.4% (04)	21.7% (05)	**0.0467**^b^
	Rivne	50.0% (11)	82.6% (19)	78.3% (18)	
Marital status	Married or cohabiting	72.7% (16)	91.3% (21)	100.0% (23)	**0.0093** ^b^
	Single/separated/divorced	27.3% (06)	8.7% (02)	0.0% (00)	
Education	Less than high school	13.6% (03)	8.7% (02)	0.0% (00)	**0.0246** ^b^
	High school or equivalent	54.5% (12)	60.9% (14)	30.4% (07)	
	Some college or higher	31.8% (07)	30.4% (07)	69.6% (16)	
Socio-economic category (*sescat*, Hollingshead score)	55–66	9.1% (02)	8.7% (02)	17.4% (04)	**0.0315** ^a^
	40–54	13.6% (03)	17.4% (04)	43.5% (10)	
	30–39	45.5% (10)	43.5% (10)	21.7% (05)	
	20–29	4.5% (01)	26.1% (06)	17.4% (04)	
	08–19	27.3% (06)	4.3% (01)	0.0% (00)	
Gravidity	>1	54.5% (12)	34.8% (08)	56.5% (13)	0.3032^b^
	1	45.5% (10)	65.2% (15)	43.5% (10)	
Parity	>0	40.9% (09)	30.4% (07)	47.8% (11)	0.4767^b^
	0	59.1% (13)	69.6% (16)	52.2% (12)	
Body Mass Index		22.01 ± 4.55	22.75 ± 3.98	21.18 ± 3.05	0.3701^a^
Smoking status (*smokstat)*	Current Smoker	31.8% (07)	21.7% (05)	0.0% (00)	**0.0001** ^b^
	Never Smoked	22.7% (05)	21.7% (05)	95.7% (22)	
	Quit after realized pregnant	36.4% (08)	34.8% (08)	0.0% (00)	
	Quit Before Pregnancy	9.1% (02)	21.7% (05)	4.3% (01)	
Number of cigarettes per day during pregnancy		2.50 ±4.34	0.87 ±2.26	0.00 ±0.00	**0.0142** ^a^
Multi-Vitamins after enroll	No	36.4% (08)	39.1% (09)	43.5% (10)	0.2216^a^
	Yes	63.6% (14)	60.9% (14)	56.5% (13)	
Multi-vitamins pre-enroll	No	36.4% (08)	21.7% (05)	26.1% (06)	0.5392^a^
	Yes	63.6% (14)	78.3% (18)	73.9% (17)	
Gestational age at 1^st^ blood draw		19.09 ± 5.18	18.30 ± 5.82	18.00 ± 4.45	0.7241^a^
Gestational age at 2^nd^ blood draw		32.95 ± 2.77	33.52 ± 2.50	32.26 ± 3.15	0.2238^a^
*AAD0*: Absolute ounces of alcohol per day at time of conception		0.69 ±0.65	0.50 ±0.27	0.00 ±0.00	**0.0001** ^a^
*AADD0*: Absolute ounces of alcohol per drinking day at time of conception		1.72 ±1.33	1.50 ±1.05	0.00 ±0.00	**0.0001** ^a^
*AADXP*: absolute ounces of alcohol per day in two weeks prior to enrollment		0.09 ±0.17	0.03 ±0.06	0.00 ±0.00	**0.0003** ^a^
*AADDXP*: Absolute ounces of alcohol per drinking day in two weeks prior to enrollment		0.55 ±0.79	0.24 ±0.35	0.00 ±0.00	**0.0002** ^a^

x ± y represents the mean ± standard deviation, (n) = sample size. P-values in **Bold** are statistically significant.

^a^ Kruskal-Wallis Rank Sum Test

^b^ Fisher's Exact Test

After delivery, data were collected on gestational age at birth, birth size and sex of the infant. Live-born infants subsequently received a study-related dysmorphological examination that was conducted by a study geneticist (LY or NZZ) with specific training in FASD. Infants were evaluted for the physical features of FASD and for growth using a standard checklist. At approximately 6 and/or 12 months of age, infants were evaluated for neurobehavioral performance by a study psychologist using the Bayley Scales of Infant Development, second edition (BSID-II), and standard scores were obtained on the Mental Developmental Index (MDI) and the Psychomotor Developmental Index (PDI) after adjustment for gestational age at delivery and standardized for infant sex (Table 2).

**Table 2:** Infant characteristics of the sample, Ukraine, 2006–2011.

Variable		HEa (N = 22)	HEua (N = 23)	UE (N = 23)	p-value
Child's Sex (*CSEX*)	Male	40.9% (09)	43.5% (10)	69.6% (16)	0.1098 ^b^
	Female	59.1% (13)	56.5% (13)	30.4% (07)	
Height < 10^th^ percentile	No	86.3% (19)	100.0% (23)	95.7% (22)	0.1177 ^b^
	Yes	13.6% (03)	0.0% (00)	4.3% (01)	
Weight < 10^th^ percentile	No	86.3% (19)	95.7% (22)	91.3% (21)	0.5129^b^
	Yes	13.6% (03)	4.3% (01)	8.7% (02)	
Occipital-Frontal Circumference < 10^th^ percentile	No	63.6% (14)	100% (23)	91.3% (21)	**0.0009** ^b^
	Yes	36.4% (08)	0% (00)	8.7% (02)	
Smooth Philtrum	No	77.3% (17)	100.0% (23)	100.0% (23)	**0.0025** ^b^
	Yes	22.7% (05)	0.0% (00)	0.0% (00)	
Thin vermilion border	No	63.6% (14)	100.0% (23)	69.6% (16)	**0.0022** ^b^
	Yes	36.4% (08)	0.0% (00)	30.4% (07)	
Palpebral Fissure <10th percentile	No	45.5% (10)	100.0% (23)	95.7% (22)	**0.0001** ^b^
	Yes	54.5% (12)	0.0% (00)	4.3% (01)	
^$^MDI 6 month		81.95 ± 13.68	96.67±5.1	93.96±9.05	**0.0005** ^a^
^#^PDI 6 month		81.14 ± 15.82	99.28±9.99	96.22±10.56	**0.0068** ^a^
MDI12 month		81.10 ± 12.46	97.16±10.17	96.17±9.55	**0.0056** ^a^
PDI 12 month		90.81±16.21	106.84±10.38	103.17±11.46	**0.0368** ^a^
Gestational Age at Delivery		37.94 ± 2.51	40.00 ± 1.10	40.03 ± 1.07	0.0876 ^a^

x ± y represents the mean ± standard deviation, (n) = sample size. P-values in **Bold** are statistically significant

^$^ Mental Developmental Index

^#^ Psychomotor Development Index

^a^ Kruskal-Wallis Rank Sum Test

^b^ Fisher's Exact Test

Infants were classified as affected (having an FASD) if they were in the moderate to heavily prenatally exposed group, and had at least two characteristic alcohol-related craniofacial features (short palpebral fissures, smooth philtrum and thin vermilion border of the upper lip) and growth deficiency, and/or neurobehavioral impairment defined as scores on the BSID-II that were more than one standard deviation (15 points) below the mean on the MDI and/or PDI adjusted for prematurity (Table 2). Study protocols were approved by Institutional Review Boards at the Lviv National Medical University, Ukraine, and the University of California San Diego and Texas A&M University in the US, and research was conducted according to the principles expressed in the Declaration of Helsinki.

### Description of the Sample

The sample for this analysis was selected based on the criteria of mother-infant pairs for whom complete data were available on maternal alcohol exposure, a dysmorphological examination and neurobehavioral testing of the infant, and the availability of maternal blood samples obtained at both enrollment and in the third trimester. The sample selected consisted of three groups. The first group represented moderate to heavily-exposed mothers with an FASD-affected child (HEa, n = 22); the second group represented moderate to heavily-exposed mothers with an apparently unaffected child, i.e., no facial features, normal head circumference and normal neurobehavioral test scores (HEua, n = 23); and low alcohol consuming or unexposed mothers with an unaffected child (UE, n = 23). By group, maternal and infant characteristics were summarized from the maternal interviews and infant examinations. Socioeconomic status (*sescat*) as measured by the Hollingshead categories was based on maternal and paternal education and occupation and was classified in category 1–5 with 1 being the highest. Pre-pregnancy body mass index (BMI) was calculated based on mother’s height and her self-reported weight prior to conception. Smoking status (*smokstat*) was classifed as ‘never smoker’, ‘ever smoker but quit before pregnancy’, ‘ever smoker but quit once recognized pregnant’, and ‘continued or current smoker in pregnancy’. Among current smokers, the average number of cigarettes per day at the time of enrollment was captured. Maternal use of multivitamin supplements was collected from interviews and classified as yes/no for vitamin use prior to enrollment, and for vitamin use after enrollment. The amount of alcohol consumed in the week around conception and in the most recent two weeks prior to enrollment was calculated by daily alcohol type and quantity and classified into four summary variables: absolute ounces of alcohol per day at the time of conception (AAD0), absolute ounces of alcohol per drinking day at the time of conception (AADD0), absolute ounces of alcohol per day in the most recent two weeks (AADXP) and absolute ounces of alcohol per drinking day in the most recent two weeks (AADDXP). Maternal demographic data are summarized in Table 1, and infant outcome measurements in Table 2.

### MiRNA analysis

Plasma sample quality control analysis and RNA isolation was performed as outlined in Methods A in S1 File. MiRNA profiles were measured using Human miRCURY LNA^™^ microRNA real-time PCR arrays (V3&4, Exiqon, Denmark), which assess 752 unique miRNAs [38, 45]. This platform has been shown in comparison tests, to detect miRNAs in biofluids with greater sensitivity and specificity than other miRNA detection methods [46]. Methods and control assays for specificity and sample contamination were performed as outlined in Methods B in S1 File.

### Placental Lactogen ELISA

Human Placental Lactogen (hPL) concentration in human plasma was quantitatively determined using a commercially available solid phase sandwich-type enzyme-linked immunoassay (hPL micro-ELISA, Leinco Technologies, Inc., St. Louis, Missouri, USA). The chromogenic reaction product was quantified spectrometrically (at 450nm, Tecan Infinite m200 with Magellan v7.2 control software, Tecan, Austria) against a recombinant hPL (0–15 μg/ml) standard curve.

### Statistical modeling

Cycle Thresholds (CTs) were determined using SDS2.4 (ABI/Life Technologies). The CT for all un-amplified (unexpressed) miRNAs was set to a value of 50. ΔCT for each miRNA in each sample was calculated as the CT_miRNA_x_—CT_Average_Expressed_miRNAs_, i.e., normalized to the average CT value for all of the expressed miRNAs in that sample. All miRNA identities in V3 and V4 Exiqon qPCR array platforms were cross-referenced to their unique mature sequence accession number assigned in miRBase (MIMAT, www.mirbase.org, [47]), before data were aggregated for statistical analyses. All subsequent data analyses were linked to MIMAT accession numbers. Data were analyzed using SPSS (V20, IBM, Armonk, New York), Gensifter^®^ analysis edition (GSEA, Geospiza/PerkinElmer, Seattle, WA) and R (V3.2.2, R Foundation for Statistical Computing, Vienna, Austria). Data were subjected to Analysis of Variance (ANOVA) or T-tests, with Benjamini and Hochberg false discovery rate (FDR) correction for multiple comparisons (α = 0.05 or 0.1). Random forest analysis (RFA, [48]), a non-parametric tree-based ensemble method of classification was implemented in the R ‘randomForest’ package (V4.6–10) as a means to predict group membership of maternal samples based on a combination of miRNAs and demographic variables (see Methods C in S1 File). As a prescreening measure, we included in the RFA model, the 5% (37 out of 752) of sampled miRNAs with the highest variance (while blinded to the class labels) in order to focus in on those miRNAs with potentially the most predictively useful differences. In order to assess the prediction performance of the RFA models, we used out-of-bag (OOB) sampling to compute the misclassification rate [49]. Candidate miRNAs derived from ANOVA (11 miRNAs that exceeded FDR-corrected P < 0.05) and from random forest analysis (top 5 predictive miRNAs) were subject to pathway overrepresentation analysis using the Ingenuity Pathway Analysis^®^ software suite (IPA, QIAGEN, see Methods D in S1 File).

## Results

### Cohort characteristics

Characteristics of the mothers and infants in each of the three groups in the sample are shown in Tables 1 and 2. Notably, maternal age, pre-pregnancy body mass index (BMI), gestational age at enrollment, gestational ages at blood draws, and the frequency of multivitamin use both before and after enrollment, were similar across all three groups. There were some differences by group in the distribution of women by site, and as expected, socioeconomic status and maternal education levels were lower, and current smoking was higher in the moderate to heavy alcohol-exposed groups compared to the low or unexposed group. Importantly, while alcohol consumption did significantly differ across groups (Table 1, Kruskal-Wallis Rank Sum Test, all P’s < 0.0003 for AAD0, AADD0, AADXP and AADDXP), post-hoc analysis showed that the HEa and HEua groups were not different from each other with respect to prenatal alcohol exposure (Mann-Whitney U, AAD0, P = 0.21; AADD0, P = 0.54; AADXP, P = 0.16; AADDXP, P = 0.093, data not shown). In addition, patterns of alcohol consumption among women who smoked did not differ between the HEa and HEua groups (data not shown). In terms of outcomes, gestational age at delivery was slightly lower in the HEa group compared to HEua and UE groups, but overall, the differences were not statistically significant, and Bayley Scales of Infant Development scores, as defined for group membership, were on average lower in the HEa group than the two unaffected groups (Table 2).

**Fig 1:**
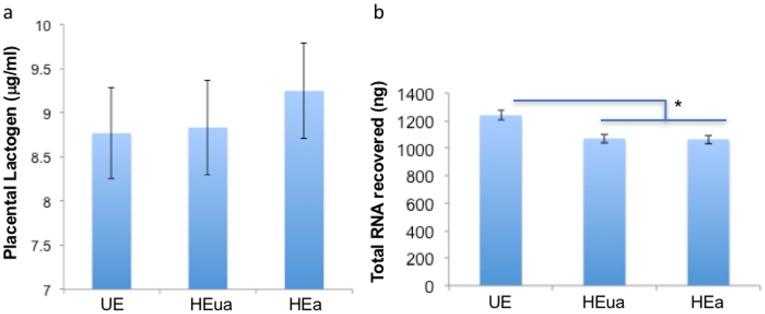
Placental Lactogen (hPL) and total RNA content in maternal plasma samples. (a) Plasma placental lactogen content in late pregnancy was not significantly different among HEa, HEua and UE groups. (b) Analysis of total plasma RNA content indicated that there was a statistically significant, ~15% decrease in total RNA recovery in the alcohol exposure groups (HEa and HEua) compared to UE controls.

All samples passed quality control analyses and showed no evidence for erythrocyte miRNA contamination (S2 File and S1 Fig). We also found no effect of exposure group on hPL levels (ANOVA, F_(2,65)_ = 0.24, P = 0.79, Fig 1a), asessed near the end of the 3^rd^ trimester. hPL is a sensitive measure of placental health [50], and consequently placental damage is unlikely to account for altered maternal miRNA profiles.

There was however, a significant effect of exposure condition on the quantity of recovered plasma total RNA (ANOVA, F_(2,124)_ = 9.86, P = 0.0001). On average 15% more total RNA was isolated from an equi-volume of plasma obtained from control mothers (UE), compared to alcohol-consuming mothers who subsequently gave birth to both affected (HEa) and unaffected (HEua) infants (all post-hoc t-test P-values < 0.05, Fig 1b). However, there was no significant effect of recruitment site (ANOVA, P = 0.41) and a marginal, though non-significant difference due to pregnancy stage (ANOVA, P = 0.051).

### Assessment of group differences in miRNA expression

We observed no group differences in total numbers of expressed miRNAs as a function of recrutiment site (P = 0.74), exposure group (P = 0.99) or pregnancy stage (P = 0.67) by ANOVA. We also did not observe significant differences in the average expression level of expressed miRNAs as a function of exposure group (P = 0.53) or pregnancy stage (P = 0.45), though there was a marginal, non-statistically-significant effect of recruitment site (ANOVA, F_(1,124)_ = 3.513, P = 0.063), on average miRNA expression. Further analysis indicated that a single miRNA, miR-29b, was significantly increased by ~200-fold in maternal samples obtained at mid-pregnancy from Khmelnytsky compared to Rivne (FDR-adjusted t-test, P = 0.047). However, no miRNAs were significantly altered as a function of recruitment site at the end of pregnancy.

**Fig 2:**
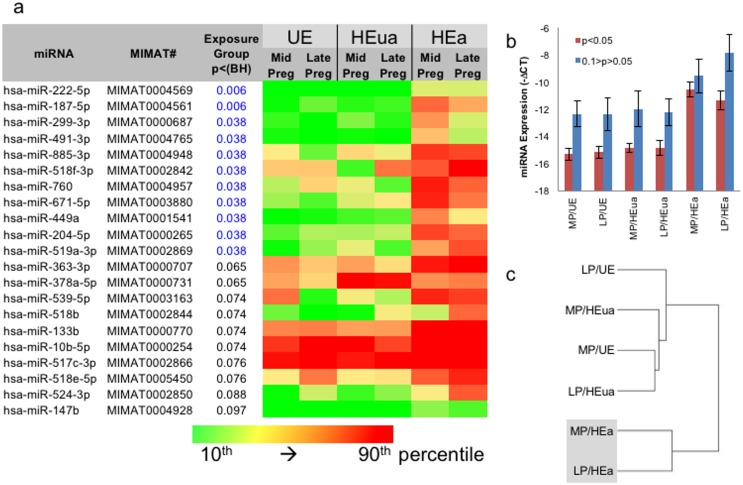
ANOVA model identifies maternal plasma miRNAs elevated specifically in the HEa group. (a) List of miRNAs that pass the FDR-corrected criterion of P < 0.05. Color scale ranges from 10^th^ (green) to 90^th^ (red) percentile of expression. miRNA expression in the HEa group at both mid and late pregnancy was generally elevated compared to expression in all other groups (for additional detail, see S2 Fig). (b) Average expression of miRNAs that exceed P < 0.05 and P < 0.1 BH-corrected criteria. (c) Cluster analysis (with Euclidean distance and average linkage) of miRNAs that exceed the BH-corrected P < 0.1 criterion indicates that HEua and UE groups cluster together and are different from HEa groups at mid and late pregnancy. MP, mid-pregnancy; LP, late pregnancy; MIMAT#######, miRNA unique ID as in mirbase.org.

In a 2-way ANOVA assessing the main effects of pregnancy stage and exposure group, and using a 2-ΔCT threshold for between-group changes as a cutoff, to eliminate smaller and perhaps non-biologically relevant group differences, we identified 11 miRNAs that exceeded a FDR of α = 0.05 and a total of 21 that exceeded α = 0.1 for the main effect of exposure condition (Fig 2a). Interestingly, a majority of significantly altered miRNAs were increased in plasma samples from women in the HEa group at both mid- and late-pregnancy compared to both HEua and UE groups (i.e., HEa>(HEua ≅ UE)). This effect was stronger for miRNAs that exceeded the FDR-corrected criterion of P < 0.05 compared to P < 0.1 (Fig 2b). Cluster analysis of miRNAs that exceeded the FDR-corrected criterion of P < 0.1 emphasized our finding that these miRNAs discriminated between the HEa group and both other groups, and that the UE control group was similar to the HEua group (Fig 2c). No miRNAs exceeded the FDR threshold of α = 0.1 for the effect of pregnancy stage.

### Random Forest Analysis and prediction of HEua group membership

The above analyses collectively show that plasma miRNA profiles can predict infant outcomes, i.e., the presence of dysmorphia and/or neurobehavioral impairment in infancy due to prenatal alcohol exposure. However, the group of prenatally exposed infants with dysmorphic features and/or neurobehavioral impairment measurable in early life are relatively easy to identify and diagnose, while it is significantly more difficult to identify the class of heavily prenatally-exposed infants who do not exhibit obvious dysmorphia or early evidence for developmental delay. To determine the extent to which members of the HEua group could be classified as more like the HEa or UE control group, we first selected the 37 miRNAs (5% of the 752 sampled miRNAs) exhibiting the highest variance irrespective of group membership. Prelimnary analyses, indicated that the 5% criteria represented the best balance between miRNA number and OOB mis-classification rate (S3 Fig). We addiitonally included demographic, clinical and other pregnancy-associated variables (maternal age, maternal multivitamin use before and after enrollment, maternal smoking status (categorical), socioeconomic status category, gravidity, parity, and infant sex) to the random forest model to predict group membership in a comparison of the HEa and UE control group.

**Fig 3:**
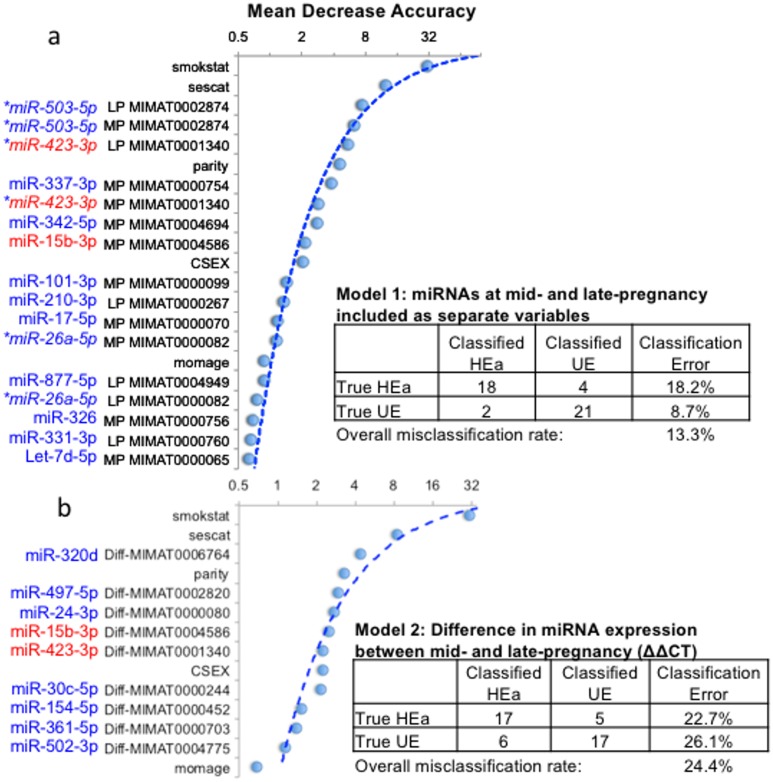
Random Forest Analysis (RFA) classifies HEa and UE maternal samples into distinct groups. (a) RFA analysis comparing HEa and UE groups at mid (MP) and late (LP) pregnancy resulted in an overall classification error rate of 13% (18.2% for the HEa group and 8.7% for the UE group). miRNAs constituted 7 out of the top 10 variables that contributed to classification accuracy. Graph depicts Mean Decrease Accuracy (the effect of permuting a variable on prediction after training) on the X-axis and contributory variables in order of decreasing importance on the Y-axis. Astrisks indicate miRNA variables that contributed to prediction accuracy at mid- and late-pregnancy. (b) RFA analysis with difference in miRNA expression (ΔΔCT) between mid and late pregnancy. The overall misclassification rate increased to 24.4%. However, a plot of ‘Mean Decrease Accuracy’ (Y-axis) against variables (X-axis) showed that miRNAs constituted 6 out of the top 10 predictive variables. miRNAs in red text represent variables present in both model 1 and 2. For additional details, see S4 Fig. Smokstat, sescat, parity and momage are as defined in Table 1, CSEX is as defined in Table 2.

Our analyses show that the HEa and UE groups can be classified into their respective groups with an overall OOB mis-classification rate (proportion of misclassified observations) of 13.3% (i.e., 6 out of 45 samples missclassified, Model 1, Fig 3a and S4 Fig). The classification model more accurately assigned membership of UE samples to the UE group, with a classification error rate of 8.7%, whereas the error rate for the HEa group was 18.2% (2 out of 23 and 4 out of 22 samples respectively, Fig 3a). Demographic variables including a history of smoking and socioeconomic status contributed heavily to the accuracy of classification. However, miRNAs, including hsa-miR-503-5p (MIMAT0002874) and hsa-miR-423-3p (MIMAT0001340) which were predictors at mid-pregnancy as well as in late pregnancy, constituted 7 out of the top 10 predictors of class membership. We next asked if a change in miRNA expression from mid- to late-pregnancy (ΔΔCT) could perform as a better classifier of class membership. However, the OOB mis-classification rate for the UE vs HEa comparison increased to 24.4% (Model 2, Fig 3b and S4 Fig), largely due to an increased mis-classification rate (26.1%) in the UE group. We earlier observed that we recovered ~15% less total RNA in HEa and HEua groups compared to UE group (Fig 1b). Total circulating RNA is heterogenous in composition and includes a number of classes of small [51]) and large RNAs [52]. Moreover, the ANOVA model identified several miRNAs that were selectively increased in the HEa group (Fig 2) but not HEua group, suggesting that this variable, total RNA, may contain additional information for class prediction, not contained in the miRNA analyses. Therefore total RNA was included in a secondary analysis. However, inclusion of the total RNA variable resulted in an overall misclassification rate of 17.78% for model 1 and to 24.44% for model 2 (S5 Fig, panels a and b), indicating that addition of this variable did not improve classification.

**Fig 4:**
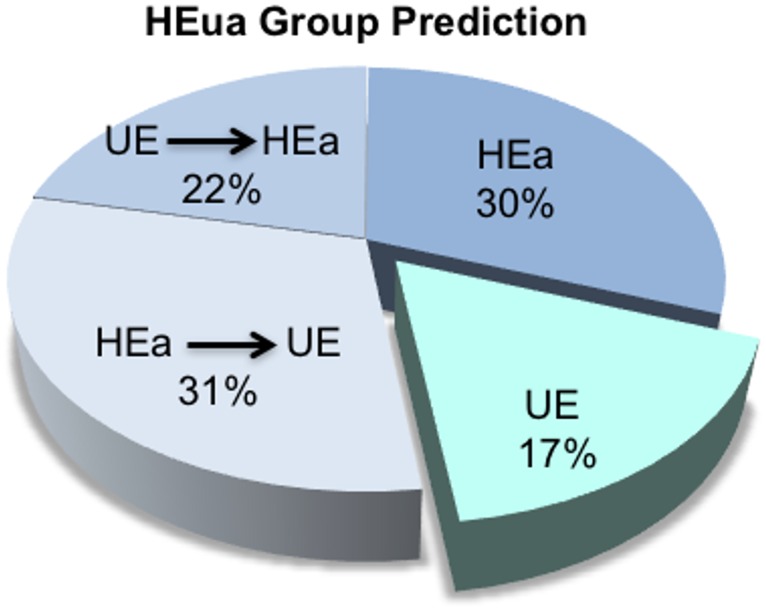
Classification of maternal samples from the HEua group. Pie chart shows classification of the HEua group as either like HEa or HEua groups. 17% of pregnant mothers assigned to the HEua group were classified as UE-like throughout pregnancy. 52% were either like the HEa (30%) throughout pregnancy or moved from being UE-like at mid pregnancy to HEa like (22%) by the end of pregnancy. However, 31% of HEua mothers were HEa-like at mid pregnancy, but more UE-like by the end of pregnancy.

We next asked whether the identified predictive variables could be used to assign members of the HEua group to HEa and UE groups. Based on the RFA model, 17% of the HEua group could be classified completely as the UE group throughout pregnancy, while 83% were more like the HEa group at some time during pregnancy. Among the HEa-like group, a majority remained stably classified as HEa-like throughout pregnancy or were identified as more HEa-like by the end of pregnancy, though a smaller sub-population of the HEa-like group moved towards the UE-like classification by the end of pregnancy (Fig 4). Including total RNA in the prediction model, 78% of the HEua group were classified as HEa-like at some time during pregnancy and 22% were classified as UE-like (S5 Fig, panel c). The total RNA-containing model resulted in increased homogeneity in the portion of the HEua group that was classified as HEa-like. In this prediction model, only 4% of the HEua group who were classified as HEa-like at mid pregnancy moved towards the UE-like classification by the end of pregnancy. Collectively these data indicate that, whereas an analysis of variance strategy emphasised the similarity of the HEua group to the UE group, the random forest classification model preferentially associated the HEua group with the HEa group, i.e., (HEa ≅ HEua)≠UE.

### Predictions of function

**Fig 5:**
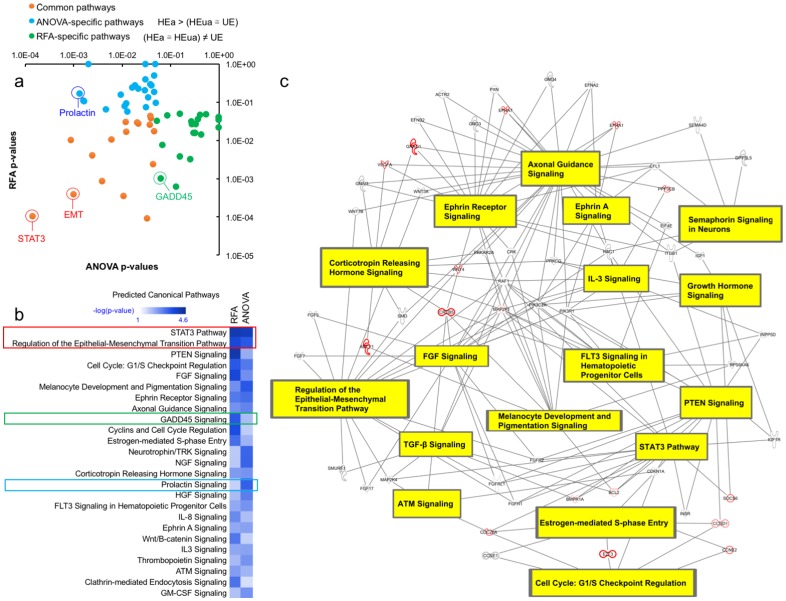
Pathway overrepresentation analysis to assess functions of predictive plasma miRNAs. Pathway overrepresentation analysis was performed using IPA software on targets of the eleven miRNAs that exceeded the FDR-corrected ANOVA criterion of P<0.05 (HEa>(HEua ≅ UE), and the five unique miRNAs among the top 10 contributory variables by random forest analysis ((HEa ≅ HEua)≠UE). (a) The -log_10_ p-values of significantly enriched pathways (P < 0.05) for both the ANOVA model and the RFA model were plotted against each other with transformed significance values for pathways exclusively enriched among the ANOVA model depicted in blue, the RFA model in green, and pathways enriched in both the RFA and ANOVA models in red. (b) A heat map was constructed of the top 25 significantly enriched pathways among the RFA and ANOVA models. (c) The 17 pathways enriched among both the RFA and ANOVA model share a high degree of interconnectedness. Proteins outlined in red indicate overlapping targeting by miRNAs in both the RFA and ANOVA models.

Plasma miRNAs may function as endocrine factors to influence recipient cells and tissues [53]. To assess potential endocrine functions, eleven miRNAs that exceeded the FDR-corrected ANOVA criterion of P < 0.05 (HEa>(HEua ≅ UE)), and the five unique miRNAs among the top 10 contributory variables by RFA ((HEa ≅ HEua)≠UE) were subject to pathway overrepresentation analysis. IPA models showed that despite non-overlapping miRNA content, both groups are predicted to influence common downstream pathways related to fetal and placental growth as well as distinct downstream pathways (Fig 5a and 5b). For example, the Stat3 and Ephrin A pathways and epithelial-mesenchymal transition (EMT) were predicted to be common targets, whereas prolactin signaling was specifically associated with the ANOVA model (HEa>(HEua ≅ UE) and the GADD45 pathway with the RFA model ((HEa ≅ HEua)≠UE). Further analysis of the common predicted pathways indicate that Ephrin, Stat3 and EMT pathways are core members of a highly interconnected and potentially coordinately regulated signaling network (Fig 5c) that is critical for placental and fetal growth and maturation.

## Discussion

FASD is a significant global health problem, but is difficult to prevent due to the combined prevalence of unplanned pregnancies and patterns of heavy alcohol consumption in women of childbearing age. The average gestational age at enrollment in our study was 18–19 weeks and many women reported alcohol use in the previous month, well into the critical period for neurogenesis and the addition of new neurons to the developing fetal brain [54]. An additional complication is that many prenatally exposed infants do not exhibit craniofacial dysmorphology or growth deficits that facilitate diagnosis. Thus in the current study population, equally heavily exposed mothers gave birth to both affected and apparently unaffected infants, i.e., HEa and HEua groups. Nevertheless, HEua offspring may exhibit later neurodevelopmental deficits that are not readily identifiable in infancy [12, 13]. Previous studies showed that circulating miRNAs in the pregnant mother are promising biomarkers for alcohol exposure in animal models [38] and in human populations [39]. We report here that plasma miRNAs in the pregnant mother may also be useful as a means to predict infant outcomes due to prenatal alcohol exposure.

The ANOVA model, which favors minimal within-group variance relative to systematic variance, selected miRNAs that were generally induced in the HEa group in both mid- and late-pregnancy, compared to HEua and UE groups, while minimizing differences between the latter two groups (HEa>(HEua ≅ UE). These miRNAs represent a maternal signature for risk in the HEa group, i.e., for giving birth to an affected infant. Moreover, this miRNA signature for prenatal ethanol effect could be observed as early as the second trimester of pregnancy and the detection of such a signature may facilitate early intervention to promote maternal-fetal health. It is likely that at least some miRNAs identified in the ANOVA model may be general indicators of risk for adverse pregnancy outcomes. For example, miR-222-5p (MIMAT0004569) which was elevated in the HEa group, is also reportedly elevated in placentas from women diagnosed with severe preeclampsia [55], which, like prenatal alcohol exposure, is an important cause of fetal intrauterine growth restriction (IUGR) in human populations [56]. Similarly, elevation of miR-299-3p (MIMAT0000687), as observed in the HEa group, has been shown to result in senescence of umbilical vein endothelial cells [57] which may also impair fetal growth. Research with animal models convincingly shows that prenatal alcohol compromises placental growth and function [58], and that these deficits correlate with fetal growth restriction [59], a characteristic of the HEa group. Therefore, one explanatory hypothesis is that the ANOVA model identified plasma miRNA in the HEa group that are biomarkers for functional insufficiency of the placenta and associated structures, which in turn increase the risk for adverse infant outcomes.

The ANOVA model did not help identify alcohol-exposed pregnancies that did not result in immediately obvious adverse infant outcomes. To better classify the HEua group, we adopted the RFA classification model. High variance miRNAs (selected without attention to the source of variance) along with a history of cigarette smoking and socioeconomic status, achieved a promising classification error rate of ~13% when comparing the HEa and UE groups. MiRNAs like miR-503-5p (MIMAT0002874) and miR-423-3p (MIMAT0001340) were stable contributors to prediction accuracy in both mid- and late pregnancy, whereas miR-337-3p (MIMAT0000754) contributed to prediction accuracy in late pregnancy. Importantly, using this model, we were able to segregate the HEua group into two sub-populations. The majority sub-population (83%) was more like the HEa group either throughout, or at one stage of pregnancy, while a smaller sub-population (17%) was more like the UE group consistently throughout pregnancy. Interestingly, a sub-population of the group classified as HEa-like at mid-pregnancy were predicted to be more like the controls by the end of pregnancy. This shift in classification suggests that currently unknown genetic or environmental resiliency factors may protect some pregnancies against adverse outcomes. It will be important to track neurocognitive performance in subcategories of the HEua group, as these children grow older, to determine whether our predictive models do indeed categorize future risk. These assessments are currently being performed.

While miRNAs contributed to the overall accuracy of group classification, two important variables, smoking history and socioeconomic status contributed significantly to the accuracy of classification of HEa and UE groups. The relationship between high variance miRNAs and smoking history or socioeconomic status is unknown at this time, but clearly, more research is needed to understand why these factors collectively contribute to predicting outcomes. Ongoing smoking during pregnancy may be a proxy measure for ongoing heavy alcohol consumption. However, cigarette smoking is also a well-established causal factor for fetal growth restriction [60, 61] and therefore, concurrent smoking may increase the severity of effects due to prenatal alcohol exposure. We also previously reported that both alcohol and nicotine target common miRNAs [20, 62]. Therefore, the predictive strength of maternal plasma miRNAs for infant outcomes may in part be determined by the history of smoking. Since 5 out of 7 members (71%) of the HEua group who were classified as HEa-like in the second trimester but as UE-like in the third trimester reported quitting smoking upon realization of pregnancy, smoking cessation programs during pregnancy may independently mitigate harm due to prenatal alcohol exposure.

Socioeconomic status, the second important contributory classification variable is strongly associated with health status and is thought to encompass a variety of physical and psychosocial stressors [63] that are likely to adversely influence fetal growth and development during pregnancy [64], and amplify deficits due to prenatal alcohol exposure [65]. Moreover, stress has also been show to alter circulating miRNA expression profiles [66], and consequently, socioeconomic status may also contribute to the alterations in miRNA expression observed in the current study. It is encouraging to note that interventions such as social enrichment, which may be expected to mitigate effects of socioeconomic status, have recently been shown to reverse effects of prenatal ethanol exposure on miRNA expression profiles [67]. Both socioeconomic status and current smoking have been identified as risk factors for FASD in other populations as well [68]. As with smoking cessation, strategies to minimize the effects of socioeconomic status may also minimize the effects of prenatal alcohol exposure.

At this time, we do not know which tissues and cell-types contribute circulating miRNAs that discriminate between the HEa and UE groups or permit classification of HEua group members. Plasma miRNAs are likely to represent a composite of cellular secretory activity and cell death mechanisms (reviewed in [69]), and while currently a controversial concept, these miRNAs may also constitute a novel class of endocrine factors that regulate protein translation in recipient cells [70–72]. The fetus may also contribute functional miRNAs to maternal circulation. For example, fetal cell-free RNA molecules appear in maternal plasma early during pregnancy and are maintained throughout pregnancy [73, 74], and trophoblast-secreted miRNAs have been shown to influence target gene expression in maternal immune cells [75]. Irrespective of their tissue source, the identified miRNAs are predicted to control important biological processes associated with fetal growth. For example, though third trimester placental lactogen levels were not altered in our study, we identified downstream prolactin signaling, which regulates angiogenesis [76] and maternal insulin metabolism [77] during pregnancy, as a candidate HEa group-specific miRNA-targeted pathway. In contrast, the Growth Arrest and DNA Damage-inducible 45 (GADD45) pathway, a pro-apoptotic pathway that is elevated in response to environmental stress [78] was preferentially identified as a candidate pathway in RFA model which emphasized commonalties between HEa and HEua groups. Despite non-overlapping miRNA content, both HEa>(HEua ≅ UE) and (HEa ≅ HEua)≠UE class miRNAs are nevertheless predicted to target a highly interconnected set of cytokine and growth factor pathways including Stat3 and Ephrin signaling pathways that converge on the epithelial-to-mesenchymal transition (EMT) process. The growth and maturation of the placenta is understood to be an EMT-like process [79] and placental angiogenesis and trophoblast invasion is mediated by activation of Stat3 [80] and ephrin [81] pathways. Collectively, our predictive models suggest that HEa>(HEua ≅ UE) and (HEa ≅ HEua)≠UE class maternal miRNAs are likely to both serve as biomarkers and functional mediators for important fetal developmental pathways.

In this pilot study, we present evidence that plasma miRNA profiles in the pregnant mother do predict alcohol-related infant health outcomes and help classify prenatal alcohol-exposed infants who are at risk for intellectual disability. Importantly, demographic variables like smoking history and socioeconomic status contribute significantly to the accuracy of risk assessment, but may also be modifiable causal factors and targets for perinatal intervention. However, these data should be viewed as preliminary and with caution. It will be important to assess whether maternal miRNA profiles discriminate between FASD and, in some respects phenotypically similar syndromes, including Williams and 22q11 deletion syndromes that have a clear genetic etiology, but also result in intellectual disability. The role of geography, ethnicity and other factors in the biofluid miRNA response to alcohol exposure during pregnancy also require further investigation. In this context, a recent study on the effects of alcohol consumption on serum (rather than plasma) miRNAs in a population of pregnant women in New Mexico [39] identified a markedly different group of alcohol-responsive miRNAs. The intent of that study was to assess miRNAs as biomarkers of exposure rather than outcome, and assessment platforms (microarray hybridization vs. qRT-PCR), biofluid source (serum vs. plasma), geography and population ethnicity all differed from the current study, and likely contributed to differences in outcomes. However, since platelets release miRNAs during the coagulation cascade [70], serum and plasma differ in miRNA content even when obtained at the same time, from the same patient [82]. While other intervening variables cannot be discounted, and require further investigation, it is possible that the New Mexico study uncovered an important effect of maternal alcohol exposure on acute hemostasis, whereas our study uncovered a more persistent, stable miRNA response to the effects of alcohol exposure. However, the results of both studies also leave open the possibility that the miRNAs that are significantly altered by alcohol exposure in the pregnant woman may not be the same as those which predict infant outcomes. Finally, several studies have shown that secreted miRNAs are biologically functional [70–72]. Therefore, maternal circulating miRNAs may also be targeted in future for therapeutic intervention, to promote fetal growth and development, especially in pregnancies that are predicted to result in adverse infant outcomes.

## Supporting Information

S1 File Supporting methods. (PDF)

S2 File Supporting results. (PDF)

S1 Fig Sample quality control analysis. (PDF)

S2 Fig ANOVA model data. (PDF)

S3 Fig Misclassification error as a function of the proportion of high-variance miRNAs included in RFA models. (PDF)

S4 Fig Significant variables in RFA models 1 and 2. (PDF)

S5 Fig RFA prediction models with the inclusion of the variable ‘total RNA’. (PDF)
