# Supplementary material for: Plasma miRNA Profiles in Pregnant Women Predict Infant Outcomes following Prenatal Alcohol Exposure
Source: PLoS One. 2016 Nov 9;11(11):e0165081. doi: 10.1371/journal.pone.0165081 (PMC5102408; doi:10.1371/journal.pone.0165081)
Supplement: S1 File — (PDF) [file pone.0165081.s001.pdf]

S1 File: Supporting Methods

A. Hemoglobin content analysis and RNA isolation: Plasma hemoglobin content was analyzed by NanoDrop® ND-1000 UV-Vis Spectrophotometer (Thermo Scientific, MA) for the presence of oxy-hemoglobin (500 and 600 nm) and deoxy-hemoglobin (~550nm) as evidence for erythrocyte contamination [1] and free hemoglobin (~414nm) as evidence for hemolysis, [2], as we previously published [3]. RNA was extracted from 200 μl of plasma using the RNeasy Mini kit (Qiagen, #74106). Sample yield and purity were evaluated with a NanoDrop ND-1000 spectrophotometer. A260/A280 ratios between 1.96 and 2.04 were obtained for all samples of isolated RNA, confirming purity. RNA samples were stored at −80°C before use.

B. qRT-PCR Methods. cDNA was synthesized from 25ng total RNA using the miRCURY LNA Universal RT cDNA synthesis kit, and a 1:1 mix of cDNA template and SYBR Green master mix was loaded on to PCR plates for real time, qPCR analysis on an Applied Biosystems 7900HT fast Real-time PCR system (ABI/Life Technologies, Grand Island, NY) as detailed in our publications [3, 4]. qRT-PCR Controls: (i) In samples, the *c. elegans*-specific miRNA, cel-miR-39-3p, and blank control wells did not amplify as expected. (ii) There were no statistically significant effects of recruitment site ($P<0.65$), pregnancy stage ($P<0.61$) or exposure group ($P<0.91$) on amplification of spiked-in UniSp3 controls indicating that there were no variable-specific reverse transcriptase inhibitory effects [5]. (iii) QPCR analysis for the mRNA for erythrocyte-specific band-3 membrane protein (SLC4A1) was conducted to assess erythrocyte contamination of all plasma samples using published [6] forward (5′-aacgagtgggaacgtagctg-3′) and reverse (5′-cttcatattcctcctgctccag-3′) primers. No plasma sample tested positive for SLCA4A1, though all concurrently assessed erythrocyte samples tested positive.

C: Random forest analysis (RFA, [7]), a non-parametric tree-based ensemble method of classification useful for the analysis of high-dimensional, small sample size data, was implemented in the R 'randomForest' package (V4.6-10) as a means to predict group membership of maternal samples based on a combination of miRNAs and demographic variables. RFA builds an ensemble of classification trees where each tree is constructed from a bootstrap resampling of the original dataset and at each split a random subset of features is chosen as the candidate set [8]. Averaging the low bias (each individual tree is unpruned) but high variance trees that are uncorrelated (bootstrap resampling and random feature selection produce trees that are low in correlation) creates a classifier that is both low bias and low variance [9]. One particularly advantageous aspect of RFA is the ability to rank variables in order of importance in classifying outcomes. One variable at a time is randomly permuted while the others are held fixed. This new dataset (with one variable essentially replaced with random noise) is now used for prediction and the decrease in accuracy is recorded. Those variables that, when permuted, lead to large decreases in prediction accuracy are deemed more important than variables that do not lead to large decreases. In order to assess the prediction performance of the RFA models, we used out-of-bag (OOB) sampling to compute the misclassification rate. Since each tree is built using a bootstrap resampling of the data,

the OOB sample is approximately 1/3 of the training data and was not used for the construction of the tree. This OOB sample can then be used as a test set to assess the prediction performance and misclassification rate for individual trees. We obtain a prediction for each subject using only the trees in which that subject was OOB, and can arrive at a statisticall valid estimate of the overall test-set misclassification rate [10]

D: Ingenuity Pathway Analysis. IPA miRNA Target Filter® was used to identify experimentally validated miRNA-target gene interactions or potential miRNA gene targets with a high predicted confidence of interaction (context score < -0.4 [11, 12]). IPA's Core Analysis workflow was used to conduct functional network analysis, to identify gene regulatory networks overrepresented amongst predicted miRNA targets. Specifically, overrepresentation for the ANOVA and RFA group of miRNA targets among Ingenuity's curated canonical pathways was determined based on the pathway enrichment of these targets against a reference data set consisting of the total targets of all 752 surveyed miRNAs in our assay, to control for experimental and literature biases in pathway overrepresentation. Significance of pathway overrepresentation was calculated using the Fisher's Exact Test, with $P<0.05$. Comparison of significantly enriched pathways amongst the two input groups of miRNAs was performed using the IPA Core Compare Analysis function. Visual representation of the network interaction between significantly enriched pathways was generated using IPA Path Designer.

References:

1. Zijlstra WG, Buursma A. Spectrophotometry of Hemoglobin: Absorption Spectra of Bovine Oxyhemoglobin, Deoxyhemoglobin, Carboxyhemoglobin, and Methemoglobin. Comparative Biochemistry and Physiology Part B: Biochemistry and Molecular Biology. 1997;118(4):743-9. doi: http://dx.doi.org/10.1016/S0305-0491(97)00230-7.
2. Kirschner MB, Kao SC, Edelman JJ, Armstrong NJ, Vallely MP, van Zandwijk N, et al. Haemolysis during sample preparation alters microRNA content of plasma. PLoS One. 2011;6(9):e24145. Epub 2011/09/13. doi: 10.1371/journal.pone.0024145. PubMed PMID: 21909417; PubMed Central PMCID: PMC3164711.
3. Balaraman S, Lunde ER, Sawant O, Cudd TA, Washburn SE, Miranda RC. Maternal and neonatal plasma microRNA biomarkers for fetal alcohol exposure in an ovine model. Alcohol Clin Exp Res. 2014;38(5):1390-400. Epub 2014/03/05. doi: 10.1111/acer.12378. PubMed PMID: 24588274; PubMed Central PMCID: PMC3999266.
4. Selvamani A, Williams MH, Miranda RC, Sohrabji F. Circulating miRNA profiles provide a biomarker for severity of stroke outcomes associated with age and sex in a rat model. Clin Sci (Lond). 2014;127(2):77-89. Epub 2014/01/17. doi: 10.1042/CS20130565. PubMed PMID: 24428837.
5. Blondal T, Jensby Nielsen S, Baker A, Andreasen D, Mouritzen P, Wrang Teilum M, et al. Assessing sample and miRNA profile quality in serum and plasma or other biofluids. Methods. 2013;59(1):S1-6. Epub 2012/10/06. doi: 10.1016/j.ymeth.2012.09.015. PubMed PMID: 23036329.
6. Perrotta S, Borriello A, Scaloni A, De Franceschi L, Brunati AM, Turrini F, et al. The N-terminal 11 amino acids of human erythrocyte band 3 are critical for aldolase

binding and protein phosphorylation: implications for band 3 function. Blood. 2005;106(13):4359-66. Epub 2005/08/25. doi: 10.1182/blood-2005-07-2806. PubMed PMID: 16118313.
7. Breiman L. Random Forests. Machine Learning. 2001;45(1):5-32. doi: 10.1023/a:1010933404324.
8. Chen X, Ishwaran H. Random forests for genomic data analysis. Genomics. 2012;99(6):323-9. Epub 2012/05/02. doi: 10.1016/j.ygeno.2012.04.003. PubMed PMID: 22546560; PubMed Central PMCID: PMC3387489.
9. Diaz-Uriarte R, Alvarez de Andres S. Gene selection and classification of microarray data using random forest. BMC bioinformatics. 2006;7:3. Epub 2006/01/10. doi: 10.1186/1471-2105-7-3. PubMed PMID: 16398926; PubMed Central PMCID: PMC1363357.
10. James J, Witten D, Hastie T, Tibshirani R. An Introduction to Statistical Learning with Applications in R. DeVeaux R, Fienberg SE, editors. New York: Springer-Verlag; 2015.
11. Grimson A, Farh KK, Johnston WK, Garrett-Engele P, Lim LP, Bartel DP. MicroRNA targeting specificity in mammals: determinants beyond seed pairing. Mol Cell. 2007;27(1):91-105. Epub 2007/07/07. doi: 10.1016/j.molcel.2007.06.017. PubMed PMID: 17612493.
12. Garcia DM, Baek D, Shin C, Bell GW, Grimson A, Bartel DP. Weak seed-pairing stability and high target-site abundance decrease the proficiency of lsy-6 and other microRNAs. Nature structural & molecular biology. 2011;18(10):1139-46. Epub 2011/09/13. doi: 10.1038/nsmb.2115. PubMed PMID: 21909094; PubMed Central PMCID: PMC3190056.
