# Supplementary material for: Plasma miRNA Profiles in Pregnant Women Predict Infant Outcomes following Prenatal Alcohol Exposure
Source: PLoS One. 2016 Nov 9;11(11):e0165081. doi: 10.1371/journal.pone.0165081 (PMC5102408; doi:10.1371/journal.pone.0165081)
Supplement: S2 File — (PDF) [file pone.0165081.s002.pdf]

S2 File: Supplementary Data:

Sample Q/C analysis. Erythrocyte lysis is an important source of miRNA contamination in plasma samples [1, 2]. Therefore, as published previously [3], we assessed the quality of the plasma samples and derived RNA in four ways. Firstly, we assessed plasma samples for free hemoglobin content as well as bound oxy- and de-oxy hemoglobin by spectrometric analysis. We found no evidence for whole erythrocyte contamination (i.e., no detectable oxy and deoxyhemoglobin **S1 Fig., a**) in plasma samples, and minimal hemolysis (spectral absorbance at 414nm), equal to or less than previously published [3]. There was no significant effect of recruitment site (P=0.88) or exposure group (P=0.76) on hemolysis. Interestingly, there was a small but statistically significant decline in free hemoglobin at the end of pregnancy compared to the mid-pregnancy period (ANOVA, $F_{(1,125)}=6.67$, $P<0.01$, **S1 Fig., b**), indicative perhaps of pregnancy-associated anemia in this cohort. Secondly, none of the assessed plasma samples amplified the mRNA transcript for Band-3 protein (SLC4A1, **S1 Fig., c**) which is abundent in erythrocytes. Thirdly, the mean sample ΔCq for $_{\text{plasma_stable}}$miR23a-$_{\text{erythrocyte_abundent}}$miR451a, -0.88 ± 0.677 (Mean ± SEM), was below the identified threshold for hemolysis (ΔCq>7, [4], and was not significantly altered due to recruitment site (P=0.94), pregnancy stage (P=0.71) or exposure group (P=0.65) indicating that there was not a systematic variable-dependent effect on hemolysis. These data collectively eliminate hemolysis as a signifciant contributor to sample miRNA content.

References:

1. Kirschner MB, Kao SC, Edelman JJ, Armstrong NJ, Vallely MP, van Zandwijk N, et al. Haemolysis during sample preparation alters microRNA content of plasma. PLoS One. 2011;6(9):e24145. Epub 2011/09/13. doi: 10.1371/journal.pone.0024145. PubMed PMID: 21909417; PubMed Central PMCID: PMC3164711.

2. Pritchard CC, Kroh E, Wood B, Arroyo JD, Dougherty KJ, Miyaji MM, et al. Blood cell origin of circulating microRNAs: a cautionary note for cancer biomarker studies. Cancer Prev Res (Phila). 2012;5(3):492-7. Epub 2011/12/14. doi: 10.1158/1940-6207.CAPR-11-0370. PubMed PMID: 22158052.

3. Balaraman S, Lunde ER, Sawant O, Cudd TA, Washburn SE, Miranda RC. Maternal and neonatal plasma microRNA biomarkers for fetal alcohol exposure in an ovine model. Alcohol Clin Exp Res. 2014;38(5):1390-400. Epub 2014/03/05. doi: 10.1111/acer.12378. PubMed PMID: 24588274; PubMed Central PMCID: PMC3999266.

4. Blondal T, Jensby Nielsen S, Baker A, Andreasen D, Mouritzen P, Wrang Teilum M, et al. Assessing sample and miRNA profile quality in serum and plasma or other biofluids. Methods. 2013;59(1):S1-6. Epub 2012/10/06. doi: 10.1016/j.ymeth.2012.09.015. PubMed PMID: 23036329.
