# Supplementary material for: Plasma miRNA Profiles in Pregnant Women Predict Infant Outcomes following Prenatal Alcohol Exposure
Source: PLoS One. 2016 Nov 9;11(11):e0165081. doi: 10.1371/journal.pone.0165081 (PMC5102408; doi:10.1371/journal.pone.0165081)
Supplement: S1 Fig — (PDF) [file pone.0165081.s003.pdf]

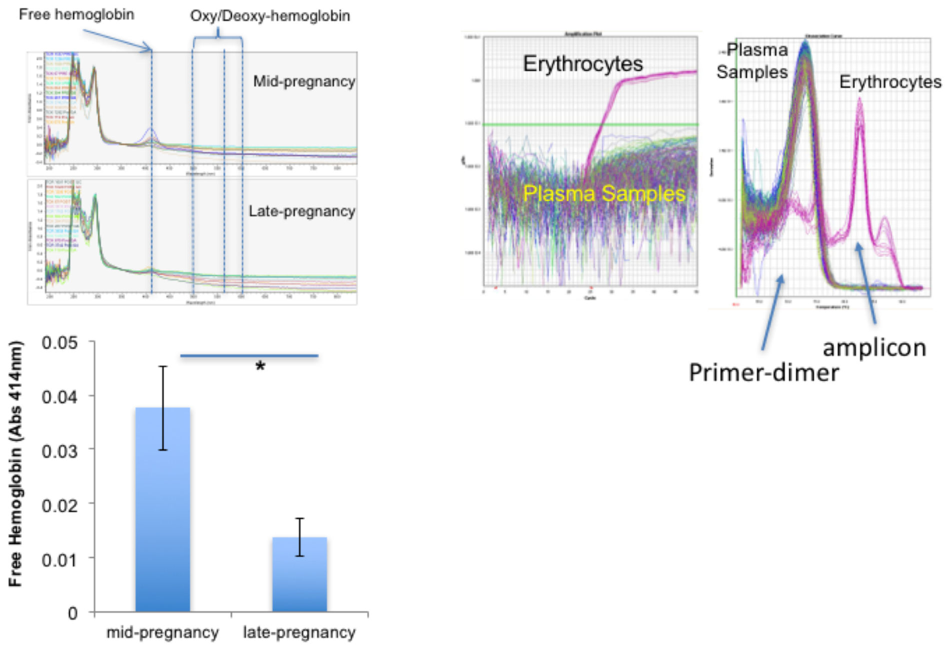

S1 Fig.

Supporting Figure S1:

S1 Fig: (a) Absorbance spectra for plasma samples obtained during mid- and late-pregnancy showed the presence of a small amount of free hemoglobin, particularly in mid pregnancy, but no evidence for contamination by erythrocytes (oxy and deoxy hemoglobin). (b) There was a small, but statistically significant decrease in free hemoglobin in late pregnancy compared to mid-pregnancy. (c) qRT-PCR analysis of RNA samples obtained from erythrocytes showed abundant expression of mRNA transcript for Band-3 protein (SLC4A1), but no expression was observed in any of the assessed plasma samples, indicating that erythrocyte lysis was not significant in these samples. c.i. shows amplification profiles, while c.ii shows sample thermal stability profiles. The left-shifted peak (c.ii) in plasma samples indicates primer-dimerization and absence of an amplicon in those samples.
