# Supplementary material for: Plasma miRNA Profiles in Pregnant Women Predict Infant Outcomes following Prenatal Alcohol Exposure
Source: PLoS One. 2016 Nov 9;11(11):e0165081. doi: 10.1371/journal.pone.0165081 (PMC5102408; doi:10.1371/journal.pone.0165081)
Supplement: S2 Fig — (PDF) [file pone.0165081.s004.pdf]

p-value (B&H Corrected)			Mean ΔCT						SEM ΔCT							
			UE		HEua		HEa		UE		HEua		HEa			
Pregnancy Stage	Exposure Group	Interaction	Mid preg	Late preg	Mid preg	Late preg	Mid preg	Late preg	Mid preg	Late preg	Mid preg	Late preg	Mid preg	Late preg	miRNA	MIMAT#
0.958	0.006	0.981	16.87	16.77	16.37	16.89	13.92	13.97	0.32	0.25	0.61	0.29	1.10	0.99	hsa-miR-222-5p	MIMAT0004569
0.958	0.006	0.871	16.21	15.16	15.87	15.62	10.28	11.89	0.65	1.02	0.91	0.93	1.54	1.43	hsa-miR-187-5p	MIMAT0004561
0.658	0.038	0.926	15.70	16.77	14.88	15.88	11.39	13.99	0.82	0.25	1.02	0.72	1.52	1.05	hsa-miR-299-3p	MIMAT0000687
0.686	0.038	0.947	16.07	16.77	16.17	16.89	12.45	14.26	0.78	0.25	0.70	0.29	1.46	1.19	hsa-miR-491-3p	MIMAT0004765
0.815	0.038	0.987	13.54	15.17	13.06	13.72	9.00	9.46	1.26	0.99	1.32	1.13	1.85	1.80	hsa-miR-885-3p	MIMAT0004948
0.658	0.038	0.851	12.71	12.65	15.59	10.57	9.86	6.90	1.33	1.36	0.82	1.49	1.45	1.33	hsa-miR-518f-3p	MIMAT0002842
0.866	0.038	0.871	14.31	12.90	13.75	15.34	8.33	10.16	1.23	1.65	1.34	1.13	1.77	1.69	hsa-miR-760	MIMAT0004957
0.846	0.038	0.926	14.57	15.42	13.94	13.45	8.52	10.67	1.23	0.99	1.38	1.50	1.76	1.65	hsa-miR-671-5p	MIMAT0003880
0.860	0.038	0.851	16.87	16.07	15.56	15.33	11.00	13.57	0.32	0.72	0.95	1.01	1.64	1.30	hsa-miR-449a	MIMAT0001541
0.966	0.038	0.961	15.31	14.39	14.45	14.17	9.34	10.25	1.03	1.32	1.31	1.35	1.83	1.74	hsa-miR-204-5p	MIMAT0000265
0.860	0.038	0.851	16.20	14.66	13.79	15.48	11.74	9.62	0.74	1.09	1.39	1.03	1.64	1.68	hsa-miR-519a-3p	MIMAT0002869
0.958	0.065	0.851	11.37	12.66	11.67	13.18	8.25	4.56	1.84	1.70	1.87	1.61	2.11	2.04	hsa-miR-363-3p	MIMAT0000707
0.864	0.065	0.960	11.77	12.96	6.72	7.98	11.28	10.85	1.46	1.31	1.47	1.56	1.42	1.52	hsa-miR-378a-5p	MIMAT0000731
0.658	0.074	0.851	10.48	15.91	13.65	14.37	8.56	9.21	1.80	0.81	1.43	1.39	1.95	1.81	hsa-miR-539-5p	MIMAT0003163
0.658	0.074	0.851	15.10	16.77	16.14	13.63	14.14	10.25	1.10	0.25	0.73	1.34	1.19	1.52	hsa-miR-518b	MIMAT0002844
0.846	0.074	0.928	11.01	10.82	11.64	11.57	7.07	4.07	1.79	1.94	1.78	1.83	2.00	1.92	hsa-miR-133b	MIMAT0000770
0.958	0.074	0.851	8.98	6.27	5.29	9.35	3.42	2.54	1.72	1.63	1.43	1.66	1.14	1.20	hsa-miR-10b-5p	MIMAT0000254
0.812	0.076	0.992	8.03	6.53	8.25	7.35	4.26	2.88	1.48	1.47	1.45	1.42	1.19	0.84	hsa-miR-517c-3p	MIMAT0002866
0.686	0.076	0.908	13.54	10.68	13.53	13.32	9.97	8.55	1.11	1.44	1.13	1.20	1.21	1.31	hsa-miR-518e-5p	MIMAT0005450
0.658	0.088	0.871	16.24	14.07	15.66	14.78	13.59	9.89	0.71	1.23	0.97	1.16	1.27	1.58	hsa-miR-524-3p	MIMAT0002850
0.955	0.097	0.948	16.87	16.75	16.99	16.89	14.76	15.43	0.32	0.26	0.29	0.29	1.24	0.73	hsa-miR-147b	MIMAT0004928

S2 Fig.

S2 Fig. Table showing mean ± SEM of ΔCT for miRNAs that exceeded the Benjamini & Hochberg (B&H) criterion of $P<0.05$ (blue) or $P<0.1$ (black) for the main effect of exposure group.
