# Supplementary material for: Plasma miRNA Profiles in Pregnant Women Predict Infant Outcomes following Prenatal Alcohol Exposure
Source: PLoS One. 2016 Nov 9;11(11):e0165081. doi: 10.1371/journal.pone.0165081 (PMC5102408; doi:10.1371/journal.pone.0165081)
Supplement: S3 Fig — (PDF) [file pone.0165081.s005.pdf]

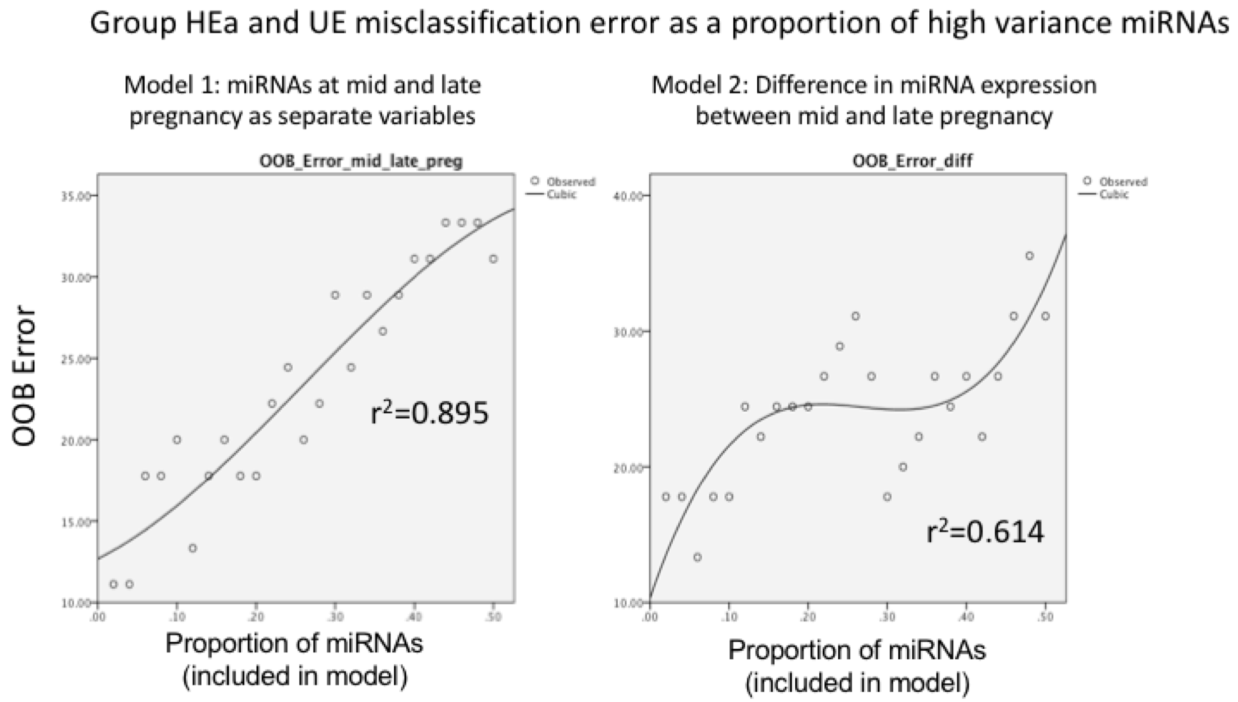

S3 Fig.

<u>S3 Fig.</u> Random forest analysis with different proportions (ranging from 2 to 50%) of high variance miRNAs irrespective of class membership. Graphs show the relationship between the out-of-bag (OOB) error rate and the proportion of high variance miRNAs included in the model. In Model 1, miRNAs from mid and late pregnancy were included as separate variables. In Model 2, the difference in miRNA expression between mid and late pregnancy (ΔΔCT) was included.
