# Supplementary material for: Plasma miRNA Profiles in Pregnant Women Predict Infant Outcomes following Prenatal Alcohol Exposure
Source: PLoS One. 2016 Nov 9;11(11):e0165081. doi: 10.1371/journal.pone.0165081 (PMC5102408; doi:10.1371/journal.pone.0165081)
Supplement: S4 Fig — (PDF) [file pone.0165081.s006.pdf]

Model 1: miRNAs at mid- and late-pregnancy included as separate variables

Variable	Mean Decrease In Accuracy
smokstat	31.2349793
sescat	12.3908923
Lp MIMAT0002874	7.44256525
Mp MIMAT0002874	6.24438558
Lp MIMAT0001340	5.5610741
parity	4.60053692
Mp MIMAT0000754	3.83730437
Mp MIMAT0001340	2.89012008
Mp MIMAT0004694	2.81044641
Mp MIMAT0004586	2.15263239
CSEX	2.09063045
Mp MIMAT0000099	1.44548284
Lp MIMAT0000267	1.35518917
Mp MIMAT0000070	1.19303071
Mp MIMAT0000082	1.14938756
momage	0.89594593
Lp MIMAT0004949	0.88702357
Lp MIMAT0000082	0.76648162
Mp MIMAT0000756	0.69939031
Lp MIMAT0000760	0.66213887
Mp MIMAT0000065	0.63706996
Lp MIMAT0000446	0.16832586
Lp MIMAT0000099	0.05203789
MIMAT0003239	0.0072464

Model2: Difference in miRNA expression between mid- and late-pregnancy (ΔΔCT)

Variable	Mean Decrease In Accuracy
smokstat	31.06393602
sescat	8.436505885
Diff-MIMAT0006764	4.405359434
parity	3.284078795
Diff-MIMAT0002820	2.965049075
Diff-MIMAT0000080	2.759856444
Diff-MIMAT0004586	2.499760109
Diff-MIMAT0001340	2.262839468
CSEX	2.260439439
Diff-MIMAT0000244	2.177098016
Diff-MIMAT0000452	1.545810412
Diff-MIMAT0000703	1.402999971
Diff-MIMAT0004775	1.13741312
momage	0.691944058
Diff-MIMAT0000762	0.12592057
Diff-MIMAT0004949	0.110602036
Diff-MIMAT0004509	0.062465412

S4 Fig.

S4 Fig. Significant variables for Random Forest Classification models. Lp, late pregnancy; Mp, Mid-pregnancy; Diff-, difference in ΔCT (change in miRNA expression) between mid and late pregnancy; smokstat, smoking status; sescat, socioeconomic status; CSEX, infant's sex; momage, maternal age at enrollment
