# Supplementary material for: Plasma miRNA Profiles in Pregnant Women Predict Infant Outcomes following Prenatal Alcohol Exposure
Source: PLoS One. 2016 Nov 9;11(11):e0165081. doi: 10.1371/journal.pone.0165081 (PMC5102408; doi:10.1371/journal.pone.0165081)
Supplement: S5 Fig — (PDF) [file pone.0165081.s007.pdf]

Models 1 & 2, including Total RNA in the Random Forest Classification Models

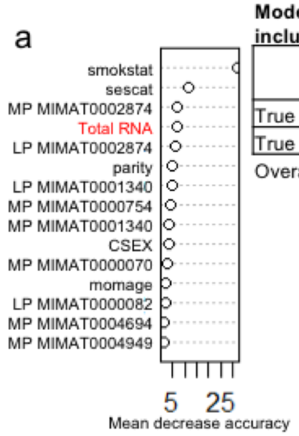

Model 1: miRNAs at mid- and late-pregnancy included as separate variables

	Classified HEa	Classified UE	Classification Error
True HEa	17	5	22.73%
True UE	3	20	13.04%
Overall misclassification rate:			17.78%

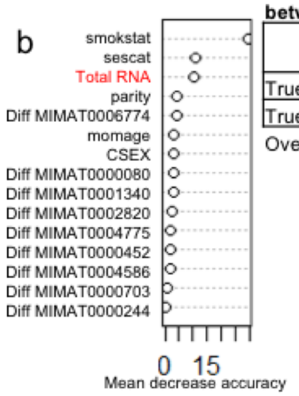

Model 2: Difference in miRNA expression between mid- and late-pregnancy (ΔΔCT)

	Classified HEa	Classified UE	Classification Error
True HEa	16	6	27.27%
True UE	5	18	21.74%
Overall misclassification rate:			24.44%

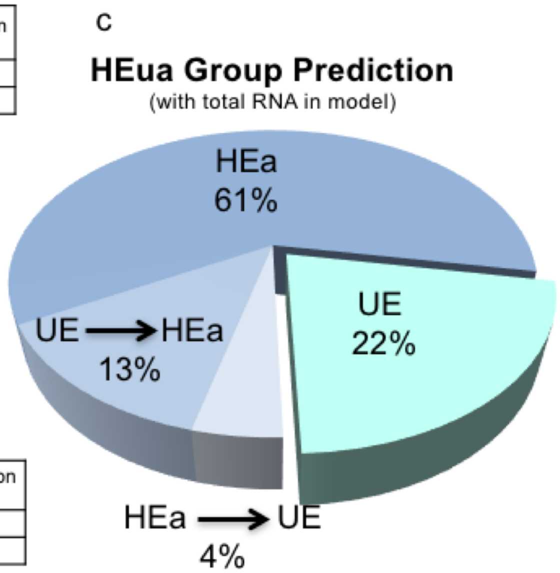

S5 Fig.

<u>S5 Fig</u>. Prediction model with the variable 'total recovered RNA' (total RNA) included. (a) Model 1 which includes miRNAs (classified by MIMAT#######) from mid-(MP) and late-(LP) pregnancy as separate variables. Overall misclassification rate was 17.78%. (b) Model 2 includes the difference in miRNA expression between mid- and late-pregnancy. Overall misclassification rate was 24.44%. (c) Using classification model #1 to classify the HEua group, results in a majority of the HEua group predicted to be HEa-like throughout pregnancy.
